# Healthcare-associated Crimean-Congo haemorrhagic fever in Turkey, 2002–2014: a multicentre retrospective cross-sectional study

**DOI:** 10.1016/j.cmi.2015.11.024

**Published:** 2016-04

**Authors:** H. Leblebicioglu, M. Sunbul, R. Guner, H. Bodur, C. Bulut, F. Duygu, N. Elaldi, G. Cicek Senturk, Z. Ozkurt, G. Yilmaz, T.E. Fletcher, N.J. Beeching

**Affiliations:** 1) Department of Infectious Diseases and Clinical Microbiology, Ondokuz Mayis University Medical School, Samsun, Turkey; 2) Department of Infectious Diseases and Clinical Microbiology, Yildirim Beyazit University Medical School, Ankara, Turkey; 3) Department of Infectious Diseases and Clinical Microbiology, Ankara Numune Research and Training Hospital, Ankara, Turkey; 4) Department of Infectious Diseases and Clinical Microbiology, Ankara Research and Training Hospital, Ankara, Turkey; 5) Department of Infectious Diseases and Clinical Microbiology, Gaziosmanpasa University Medical School, Tokat, Turkey; 6) Department of Infectious Diseases and Clinical Microbiology, Cumhuriyet University Medical School, Sivas, Turkey; 7) Department of Infectious Diseases and Clinical Microbiology, SB Diskapi Yildirim Beyazit Training and Research Hospital, Ankara, Turkey; 8) Department of Infectious Diseases and Clinical Microbiology, Ataturk University Medical School, Erzurum, Turkey; 9) Department of Infectious Diseases and Clinical Microbiology, Karadeniz Technical University Medical School, Erzurum, Turkey; 10) Liverpool School of Tropical Medicine, Liverpool, United Kingdom; 11) NIHR HPRU in Emerging and Zoonotic Infections, University of Liverpool, Liverpool, United Kingdom

**Keywords:** Crimean-Congo haemorrhagic fever, healthcare associated, infection prevention and control, ribavirin, viral haemorrhagic fever

## Abstract

Healthcare-related transmission of Crimean-Congo haemorrhagic fever (CCHF) is a well-recognized hazard. We report a multicentre retrospective cross-sectional study undertaken in Turkey in 2014 in nine hospitals, regional reference centres for CCHF, covering the years 2002 to 2014 inclusive. Data were systematically extracted from charts of all personnel with a reported health care injury/accident related to CCHF. Blood samples were tested for CCHF IgM/IgG by enzyme-linked immunosorbent assay and/or viral nucleic acid detection by PCR after the injury. Fifty-one healthcare-related exposures were identified. Twenty-five (49%) of 51 resulted in laboratory-confirmed infection, with a 16% (4/25) overall mortality. The main route of exposure was needlestick injury in 32/51 (62.7%). A potential benefit of post-exposure prophylaxis with ribavirin was identified.

Crimean-Congo haemorrhagic fever (CCHF) is a potentially fatal viral disease and a major emerging infectious disease threat after the expanding distribution of its main vector, ticks of the genus *Hyalomma* [1]. CCHF is geographically widespread across Africa, Eastern Europe, Asia and the Middle East [2] and is occasionally seen in travellers returning from endemic areas [3].

Healthcare personnel are at risk from occupational infections during patient care; the first such cases were described in Pakistan and were later reported from many Eurasian countries [4], [5], [6], [7], [8], [9]. Isolated imported cases of CCHF or outbreaks in countries lacking CCHF experience present particular infection control challenges, increased risk to healthcare workers (HCWs), and are associated with increased mortality [6]. Critical care management, associated with invasive procedures and the potential for aerosolization in highly viraemic patients, poses additional challenges [7]. The benefit and evidence for ribavirin or other post-exposure prophylaxis (PEP) options are lacking [8].

We undertook a multicentre retrospective cross-sectional study in 2014 in nine hospitals in Turkey covering the years 2002 to 2014 inclusive. These nine centres managed approximately 50% of confirmed cases of CCHF in Turkey during the study period, acting as tertiary centres for more severe and complicated disease.

We collected background data on the demographics of each hospital population and extracted demographic, epidemiologic and clinical data from the medical records of all personnel with a reported healthcare-related exposure related to CCHF. Blood samples of these healthcare personnel were tested for CCHF IgM/IgG by enzyme-linked immunosorbent assay and/or viral nucleic acid detection by PCR in regional reference laboratories. Cases without results were excluded from analysis. The outcomes were classified using a strict case definition: all confirmed HCW cases had positive serology (IgM or IgG seroconversion) and/or positive PCR results. Thirteen of the cases identified have been reported in two previous publications [8], [9]. Asymptomatic cases were confirmed by CCHF IgG seroconversion.

During the study period 9069 confirmed cases were reported nationally with a case-fatality rate of 4.5% [10], of which 4869 cases were admitted to the nine centres with a case-fatality rate of 6.7%. Fifty-one healthcare-related exposures were identified in the nine centres, with four deaths. They comprised 22 physician trainees (residents) (43.1%), 21 nurses (41.2%), two physician specialists (5.4%), two medical students (5.4%), two other ward-based staff and two laboratory technicians.

The main routes of exposure were needlestick injury (NSI) in 32/51 (62.7%), defined blood/bodily fluid exposure to mucous membranes (splash) in 12/51 (23.5%) and unidentified in 7/51 (13.7%). The exposures all occurred from cases that were subsequently confirmed to be CCHF positive by PCR. At the time of the exposure, the majority of source cases were already known to have confirmed CCHF (28/50). Ten of 50 were suspected cases, and 12/50 had not had a diagnosis of CCHF considered at the time of exposure. Related to the recipient's exposure event, 48% of the source CCHF cases died.

Overall, 25/51 (49%) had laboratory-confirmed infection (Fig. 1a). After NSI, 8/32 (25%) had laboratory-confirmed infection, 3/8 (37.5%) of whom also had clinical disease. After splash exposure, 10/12 (83.3%) had laboratory-confirmed infection, 8/10 (80%) of whom also had clinical disease. Seven cases in HCWs had no identified source of exposure. All of them had laboratory-confirmed infection and clinical signs of CCHF infection; one died. The two infections that occurred in laboratory staff were included in the unidentified exposure group, although one may have occurred while taking blood from a CCHF patient and the second while handling a blood sample in the laboratory without wearing gloves.

post-exposure ribavirin prophylaxis (oral formulation) was administered after 19/32 NSI exposures. There were no cases of clinical disease or laboratory-confirmed infection in this group. Median duration of ribavirin PEP was 7 days (range, 1–10 days), but systematic data on timing or dosage were not available. In the known exposure group that did not receive post-exposure ribavirin prophylaxis, 18/25 (68%) had laboratory-confirmed infection (8/13 in the NSI group), and 11/25 (44%) had clinical disease (Fig. 1b). In the group that received ribavirin PEP, 31.6% of the source cases died, whilst in the group that did not receive ribavirin PEP, 67.7% of the sources died.

Healthcare-related transmission of CCHF virus is dangerous, with an estimated total of 90–95 exposures of staff in 9069 admissions (∼1%) in 12 years. The risk of CCHF virus transmission in our series is 25% after a NSI. The data showing higher rates of confirmed infection after splash exposure probably reflect reporting bias, but they may be influenced by the higher rate of ribavirin PEP utilized in NSI. Clinical illness developed in 18/25 of confirmed infections, with a 22.2% mortality rate.

During the study period, an estimated minimum of 90,000–100,000 blood samples from CCHF patients were analysed in routine laboratories in the nine centres. All centres currently use modern closed or semi-closed multichannel laboratory equipment, and our data suggest that there is little hazard from processing haematology and biochemistry blood samples while following routine diagnostic laboratory procedures and using standard precautions.

Ribavirin has broad-spectrum antiviral activity, and although it is associated with a number of adverse effects, most are mild, and all are reversible. It is recommended for use in Lassa fever PEP [11]. Although to our knowledge this is the largest data set reported, the methodology and numbers are too small to draw clear conclusions on the effectiveness of ribavirin administered for CCHF PEP. An underlying bias may have influenced cases selected for ribavirin PEP, and it is not possible to adjust for other factors that may have influenced the results. For example, the case fatality rate of the source CCHF patients (causing the exposure) was lower in the group that received ribavirin PEP than in those that did not. Because of the lack of efficacy of studies to date [12], ribavirin is no longer routinely used in the treatment of CCHF in Turkey. It is possible that it is effective when there is a smaller inoculum, but it is ineffective in treating the high virus loads of clinical disease. There is no clear consensus on the ribavirin dosage for PEP, and its role can only fully be answered by well-designed multicentre controlled prospective studies.

Despite the potential availability of PEP for any viral haemorrhagic fever (VHF), the protection of all staff against nosocomial exposure and infection is paramount. Lessons in HCW protection can be learned from the ongoing Ebola virus disease epidemic, particularly in the routine utilization of personal protective equipment. Ward-based staff undertaking invasive procedures and dealing with CCHF patient contacts are most at risk. This particularly includes trainees and students, where there is high staff turnover. Advanced infection prevention and control (IP&C) training focused on sharps safety and personal protective equipment is vital for all clinical staff in endemic areas, accompanied by wider education of all HCWs, because CCHF was not initially considered in 25% of exposure cases. Every CCHF exposure, infection or death of a HCW must be considered unacceptable, avoidable and preventable.

## Transparency declaration

Financial support was received from Wellcome Trust and the UK Ministry of Defence (to TF) and the National Institute for Health Research Health Protection Research Unit (NIHR HPRU) in Emerging and Zoonotic Infections, a partnership between the University of Liverpool, Liverpool School of Tropical Medicine and Public Health England (PHE) (to NJB). The views expressed are those of the authors and not necessarily those of the Turkish Ministry of Health, the NHS, the NIHR, the Department of Health or Public Health England. All authors report no conflicts of interest relevant to this article.

## Figures and Tables

**Fig. 1:**
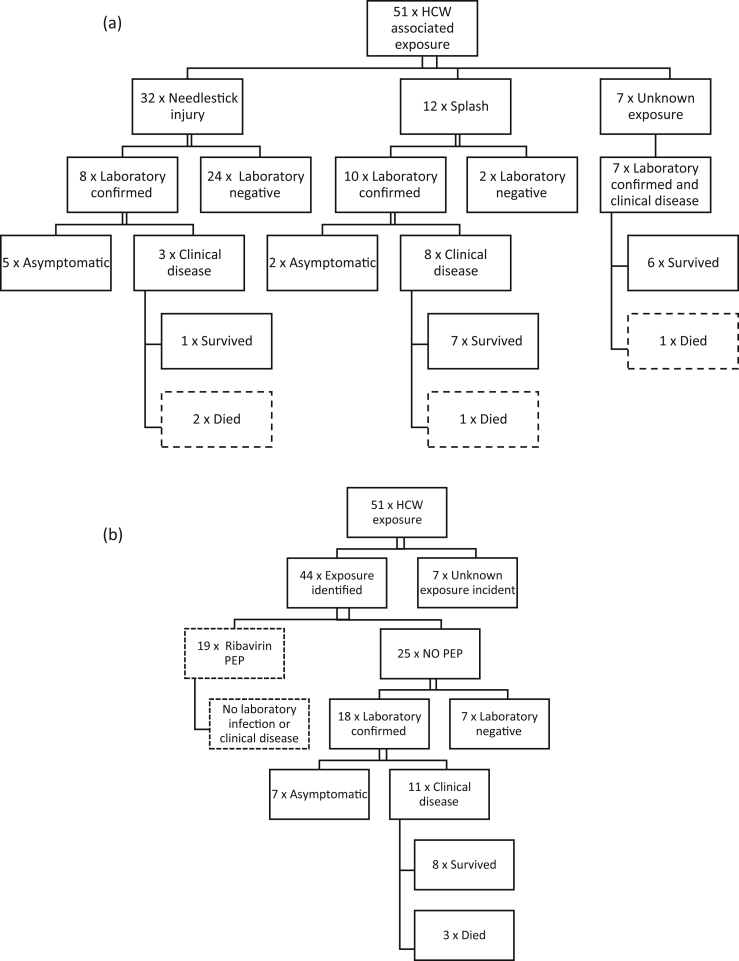
(a) Exposure and outcomes of 51 CCHF HCW exposures, related to mode of exposure and laboratory confirmation. (b) Outcome of CCHF HCW exposures stratified by administration of PEP. CCHF, Crimean-Congo haemorrhagic fever; HCW, healthcare worker; PEP, post-exposure prophylaxis.
